# In Vitro Activity and In Vivo Efficacy in a Murine Model of Rezafungin, Anidulafungin, Caspofungin, and Micafungin Against the Fifth Clade of *Candida* (*Candidozyma*) *auris*

**DOI:** 10.3390/jof12080552

**Published:** 2026-07-24

**Authors:** Dávid Balázsi, Jacques F. Meis, Jeffrey B. Locke, Gergely Udvarhelyi, Zoltán Tóth, Lajos Forgács, Csaba Kósa, Renátó Kovács, László Majoros

**Affiliations:** 1 Department of Medical Microbiology, Faculty of Medicine, University of Debrecen, Nagyerdei krt. 98, 4032 Debrecen, Hungary; 2 Doctoral School of Pharmaceutical Sciences, University of Debrecen, Nagyerdei krt. 98, 4032 Debrecen, Hungary; 3 Centre of Expertise in Mycology, Radboudumc and Canisius Wilhelmina Hospital, 6525 GA Nijmegen, The Netherlands; 4 Institute of Translational Research, Cologne Excellence Cluster on Cellular Stress Responses in Aging-Associated Diseases (CECAD), Excellence Center for Medical Mycology (ECMM), University of Cologne, 50923 Cologne, Germany; 5 Elion Therapeutics, Inc., 1 Lincoln Street, Suite 30-120, Boston, MA 02111, USA; 6 Medical Microbiology, Clinical Centre, University of Debrecen, 4032 Debrecen, Hungary; 7 Department of Surgery, Clinical Centre, Faculty of Medicine, University of Debrecen, 4032 Debrecen, Hungary

**Keywords:** *Candida auris*, fifth clade, time–kill, neutropenic mouse model, pseudohyphae production, rezafungin, echinocandins

## Abstract

**Objectives**: The aim of our study was to investigate the in vitro activity and in vivo efficacy of rezafungin, anidulafungin, caspofungin and micafungin against *Candida auris* isolates belonging to clade V. **Methods**: Five clinical isolates were evaluated (IFRC2087, IFRC4050, MRL40, TMML616 and TMML617). Echinocandin MICs, according to CLSI M27-Ed4, and killing activities were determined in RPMI-1640. In the survival and fungal tissue burden experiments (heart, kidney and brain), neutropenic mice were infected intravenously (1 × 10^7^ CFU/mouse). Treatment was initiated 24 h post-infection with intraperitoneal dosing of 20 mg/kg of rezafungin on days 1, 3 and 6 or once-daily dosing for 6 days with 3 mg/kg of caspofungin, 5 mg/kg of micafungin or 5 mg/kg of anidulafungin. **Results**: MIC ranges of rezafungin, anidulafungin, caspofungin, and micafungin were 0.06–0.25, ≤0.03–0.12, 0.12–0.5 and ≤0.03–0.12 mg/L, respectively. Growth in RPMI-1640 showed pseudohypha in all five isolates both at 30 °C and 37 °C, but hyphae were never observed. In contrast, echinocandin-treated yeasts at 37 °C showed small and large aggregates of blastoconidia at both 0.25 and 16 mg/L echinocandin concentrations. The four echinocandins at ≥1 mg/L were fungicidal only against isolate MRL40. All echinocandin regimens improved the survival in mice infected with isolates MRL40 and IFRC4050 (*p*-values were ≤0.0002 and 0.0006, respectively), but only rezafungin was effective against isolate TMML617 (*p* = 0.0049). All four echinocandins induced more than 4 log mean CFU/gram decreases in the kidneys and hearts of mice infected with isolate MRL40 (*p* < 0.001 for all echinocandins). Rezafungin and caspofungin significantly decreased the fungal kidney and heart burdens in mice infected with isolate TMML617. Against IFRC4050, rezafungin, anidulafungin and caspofungin significantly decreased the fungal burdens in the kidneys (*p* < 0.05) and hearts (*p* < 0.05–0.01). The fungal brain tissue burdens were always higher than 10^5^ CFU/gram. Histopathology showed large aggregates of pseudohyphae in the hearts, kidneys and brains in control mice. In echinocandin-treated mice, only blastoconidia were observed. **Conclusions**: In vitro activity and in vivo efficacy of the four echinocandins against the fifth clade of *C. auris* was echinocandin- and isolate-specific. Pseudohyphae production was common in controls, but not in echinocandin-treated mice. Rezafungin activity was comparable to or better than the three previously approved echinocandins.

## 1. Introduction

*Candida auris* as a globally distributed yeast is a member of the critical priority fungal pathogens according to the World Health Organization [1]. The early whole genome sequence (WGS) data of global clinical isolates indicated that four distinct clades (South Asian, East Asian, South African and South American) exist, causing hospital outbreaks and invasive infections among critically ill patients [2]. The vast majority of clinical isolates are resistant to fluconazole, and to a lesser extent to amphotericin B [3]. Current guidelines recommend echinocandins (anidulafungin, caspofungin, and micafungin) as first-line therapeutic agents to treat severe *C. auris* infections [3,4]. However, the emergence of echinocandin-resistant isolates during echinocandin treatment has been reported [5,6].

Since 2018, sporadic cases of *C. auris* infection, including meningitis, chronic mucocutaneous candidiasis and otomycoses, have been reported in Iranian patients. Although the isolate from a child suffering from meningitis belonged to the South Asian clade, the remaining isolates were genetically different from all other clades [7,8,9]. Based on WGS data, these isolates differed from the other clades by more than 200,000 single-nucleotide polymorphisms, confirming that the Iranian isolates form a separate cluster (Iranian or fifth clade). Two of five of these isolates showed high MICs to fluconazole and voriconazole, but amphotericin B, posaconazole, isavuconazole, anidulafungin and micafungin MIC values were low for all strains [10,11]. The recently identified sixth clade in Singapore and Bangladesh shows a close relationship to the South American clade [12].

Rezafungin (Rezzayo™) is the first drug approved by the FDA to treat candidemia and invasive candidiasis in more than 10 years. Rezafungin is a once-weekly, next-generation echinocandin with distinctive pharmacokinetics, including high peak concentration (>20 mg/L) and prolonged half-life (>100 h), with excellent in vitro and in vivo activity against clinically important *Candida* species. Rezafungin received approval for the treatment of candidemia and invasive candidiasis in patients older than 18 years who have limited or no alternative treatment options [13,14,15,16,17,18]. However, in cases of meningitis, endophthalmitis and urinary tract infections, or in children younger than the age of two months, amphotericin B is the first therapeutic choice [3,13]. Moreover, data on in vitro activity and in vivo efficacy of the four approved echinocandins against the Iranian *C. auris* clade are lacking.

The currently available WGS data suggest that the Iranian (fifth) clade is the closest relative of the common ancestor that diverged before the other global *C. auris* clades (I, II, III, and IV) (11). Despite the distinct phylogenetic position of the fifth clade, data are limited about its virulence or susceptibility to available antifungal agents.

The primary aim of our study was to investigate the in vitro activity of rezafungin, anidulafungin, caspofungin and micafungin using time–kill methodology against *C. auris* isolates belonging to the Iranian lineage. In addition, we determined the efficacy of the four echinocandins in a neutropenic murine model.

## 2. Materials and Methods

### 2.1. In Vitro Experiments

#### 2.1.1. Isolates

The five clinical isolates from Iranian patients [(IFRC2087 (CBS 18598), IFRS4050 (CBS 18600), MRL40 (CBS 18599), TMML616 (CBS18601) and TMML617 (CBS 18602)] used in this study are available from the CBS Westerdijk culture collection (Table 1) and were described previously [11]. None of the isolates produced aggregates in sterile physiological saline (0.9% sodium chloride). The *C. auris*-type strain (NCPF 13029 = CBS 10913; East Asian clade II) and *C. parapsilosis* ATCC 22019 were also included in MIC assays (Table 1). Two days before the experiments, isolates were sub-cultured on Sabouraud dextrose agar and screened on Candida chromogenic agar (Clinichem Kft., Budapest, Hungary, Lot number: CCA 012/110526) to ensure purity of the isolates at 35 °C [14]. All five isolates produced small, homogeneous pinkish colonies.

#### 2.1.2. Antifungal Susceptibility Testing

Anidulafungin, caspofungin and micafungin were obtained from Molcan Corporation (Richmond Hill, ON, Canada). Rezafungin was provided by Cidara Therapeutics (San Diego, CA, USA). Minimum inhibitory concentrations (MICs) were determined by the standard broth microdilution method according to CLSI (M27 Ed4) in RPMI-1640. We used round-bottomed 96-well microtiter test plates (TPP Techno Plastic Products AG, Switzerland, catalogue number 92097). Serial two-fold dilutions of the four drugs were prepared in RPMI 1640 medium (with L-glutamine, without bicarbonate; Merck (Budapest, Hungary). and buffered to pH 7.0 using a 0.165 M solution of MOPS. Antifungals were dissolved in 100% DMSO and further diluted in RPMI-1640 (Sigma, Budapest, Hungary) to final concentrations between 0.015 and 8 mg/L. The final DMSO concentration did not exceed 1% in any well. The starting inoculum was ~10^3^ CFU/mL [14]. Plates were incubated at 35 °C and MICs were read visually after 24 h [19,20,21]. The MIC was defined as the lowest drug concentration that inhibited growth to 50% of that of the drug-free control (partial inhibition criterion). MICs were determined three times. The CLSI has not yet defined breakpoints for *C. auris*. Thus, our anidulafungin, caspofungin and micafungin MICs were compared with the tentative MIC breakpoints suggested by the Centers for Disease Control and Prevention (CDC): susceptible ≤2 mg/L for both anidulafungin and micafungin, and ≤1 mg/L for caspofungin [3]. For rezafungin, a CLSI susceptible breakpoint of ≤0.5 mg/L was used [21,22].

#### 2.1.3. Phase-Contrast Microscopy

Microscopic morphology was examined in all five isolates after 24 h at 30 °C and 37 °C in RPMI-1640 in two independent experiments. Echinocandin-induced morphological alterations were determined at 0.25 and 16 mg/L with the five isolates with all four echinocandins after 24 h of incubation at 37 °C in liquid RPMI-1640 in a volume of 5 mL without agitation [19]. The starting inocula were ~10^5^ CFU/mL. A quantity of 10 µL of the suspension was gently removed from each tube (with and without echinocandins) and pipetted onto slides, and a coverslip was placed over the sample. The total number of slides examined was 20 in each experiment. In each slide, eight selected representative fields were examined. The relative frequency of pseudohyphae formation was calculated as follows: the number of cells in pseudohyphae was divided by the total number of cells per field. Pseudohyphae formation was defined as at least three (≥3) elongated fungal cells in chains. We used a Zeiss Axioskop 2 mot microscope coupled with a Zeiss Axiocam 212 camera using the phase contrast technique (Zeiss, Jena, Germany). Image acquisition was performed using Zeiss ZEN lite 3.13 software. Pseudohyphae production at 30 °C and 37 °C was compared using the Mann–Whitney U-test for each isolate.

#### 2.1.4. Time–Kill Studies

Anidulafungin, caspofungin, micafungin and rezafungin killing curves were determined with all isolates in RPMI-1640 at 0.03, 0.25, 1, 8, 16 and 32 mg/L [19]. The starting inocula were 2–3.3 × 10^5^ CFU/mL. Samples were removed at 0, 4, 8, 12 and 24 h, serially diluted tenfold, plated (4 × 30 mL) onto a single Sabouraud dextrose agar and incubated at 35 °C for 48 h. The limit of detection was 50 CFU/mL. Fungicidal or fungistatic activity was defined as ≥3 log CFU/mL or <3 log CFU/mL changes in viable cell count compared to the starting inoculum [19]. All experiments were performed twice; means of the resulting data are presented. Killing kinetics at the tested concentrations were calculated as described previously. Positive killing rate (*k*) values indicate killing and negative *k* values indicate growth. The mean times to achieve 99.9% reduction in the starting inoculum (T_99_._9_ = 3/*k*) were calculated from the *k* values for each isolate and concentration [19]. One-way ANOVA with Tukey’s post-testing was used to determine significant differences in killing kinetics among isolates and concentrations [19].

### 2.2. In Vivo Experiments

#### 2.2.1. Lethality Experiments

BALB/c female six-week-old mice (23–25 g) were given cyclophosphamide (Endoxan, Baxter, Hungary) 4 days before infection (150 mg/kg) and 1 day before infection (100 mg/kg). Immunosuppression was continued by administration of 100 mg/kg cyclophosphamide every third day until the end of the experiment on the 21st day [23]. The Guidelines for the Care and Use of Laboratory Animals were strictly followed during maintenance of the animals; experiments were approved by the Animal Care Committee of the University of Debrecen (permission no. DEMÁB 09/2024).

In order to reduce the number of mice in the in vivo experiments, isolates MRL40, IFRC4050 and TMML617 were used. We selected these isolates because TMML616 and TMML617 are from different samples of the same patient, which differed in only one SNP from each other, while IFRC4050 is fluconazole resistant [11]. Mice (groups of ten mice/isolate) were infected intravenously through the lateral tail vein (day 0). The infectious dose was 1 × 10^7^ CFU/mouse, administered in volumes of 0.2 mL. Inoculum densities were confirmed by plating serial dilutions on Sabouraud dextrose agar plates [23].

Rezafungin was dosed at 20 mg/kg on days 1, 3 and 6. The other echinocandins were dosed daily for 6 days at 3 mg/kg (caspofungin) or 5 mg/kg (micafungin and anidulafungin). All treatments were started 24 h post-infection [24]. These doses mimic the human once-weekly dosing regimen for rezafungin, and the daily dose regimens for anidulafungin, caspofungin and micafungin [24]. Untreated neutropenic control groups were given sterile physiological saline (0.9% sodium chloride). Mice were given food and water ad libitum and were monitored at least twice a day for survival for 21 days. Animals that became immobile or showed signs of severe illness were terminated and recorded as dying on the following day. Survival rates were compared using the Kaplan–Meier log rank test. Statistical tests were performed in GraphPad Prism 6.03 [24].

#### 2.2.2. Fungal Tissue Burden Experiments

The tissue bioburden experimental design was similar to that employed in the survival experiments. Each group included 6 mice. On day 7, mice were sacrificed; both kidneys, the heart and the brain were removed from each animal, weighed and homogenized aseptically in 1 mL of sterile physiological saline (0.9% sodium chloride); the resulting tissue suspension was serially diluted. Fungal tissue burden was determined by quantitative culturing. The lower limit of detection was 100 CFU/g of tissue. Mean fungal tissue burdens observed in the same organs were compared using the Kruskal–Wallis test with Dunn’s post-test [23,24].

#### 2.2.3. Histopathology

Two mice from both the treatment and control groups of the fungal tissue burden experiments were used for histopathological examination on day 7. Moreover, both on day 1 and 4, two mice infected with isolate MRL40 were used to determine the early heart, kidneys and brain involvement. Additionally, five surviving mice (control and echinocandin-treated mice) infected with isolate MRL40 from the lethality experiment on day 21 were dissected and analyzed similarly. Organs (heart, both kidneys, and brain) were fixed in 4% buffered formalin and embedded in paraffin. Tissue sections (4 µm) obtained with a microtome were stained with hematoxylin–eosin and periodic acid–Schiff [23,24].

## 3. Results

### 3.1. MIC Values of the Echinocandins Against C. auris

MICs of anidulafungin, caspofungin, and micafungin were not higher than the suggested tentative CDC breakpoints for *C. auris* (Table 1) [3]. Rezafungin MICs were lower than a provisional CLSI susceptible breakpoint (≤0.5 mg/L) [22].

### 3.2. Phase-Contrast Microscopy

**Figure 1:**
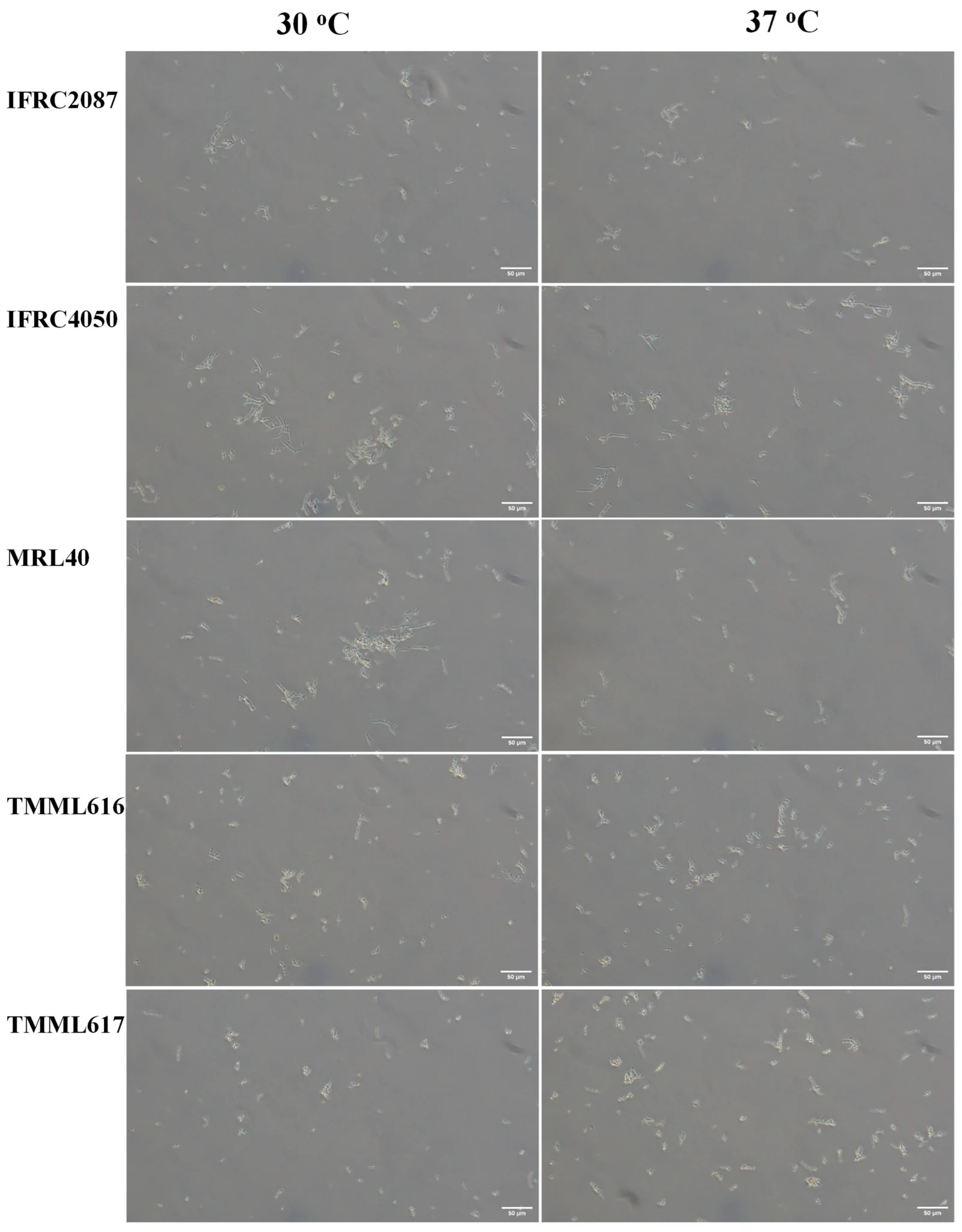
Phase-contrast microscopy images of *C. auris* cells in RPMI-1640 with isolates IFRC2087, IFRC4050, MRL40, TMML616 and TMML617 at 30 °C and 37 °C after 24 h incubation. Shorter (10–20 µm) and longer (>100 µm) pseudohyphae, and single and budding cells, were detected in all five isolates. Fungal cell aggregates were not noticed. Bar: 50 µm. Magnification, ×200.

**Figure 2:**
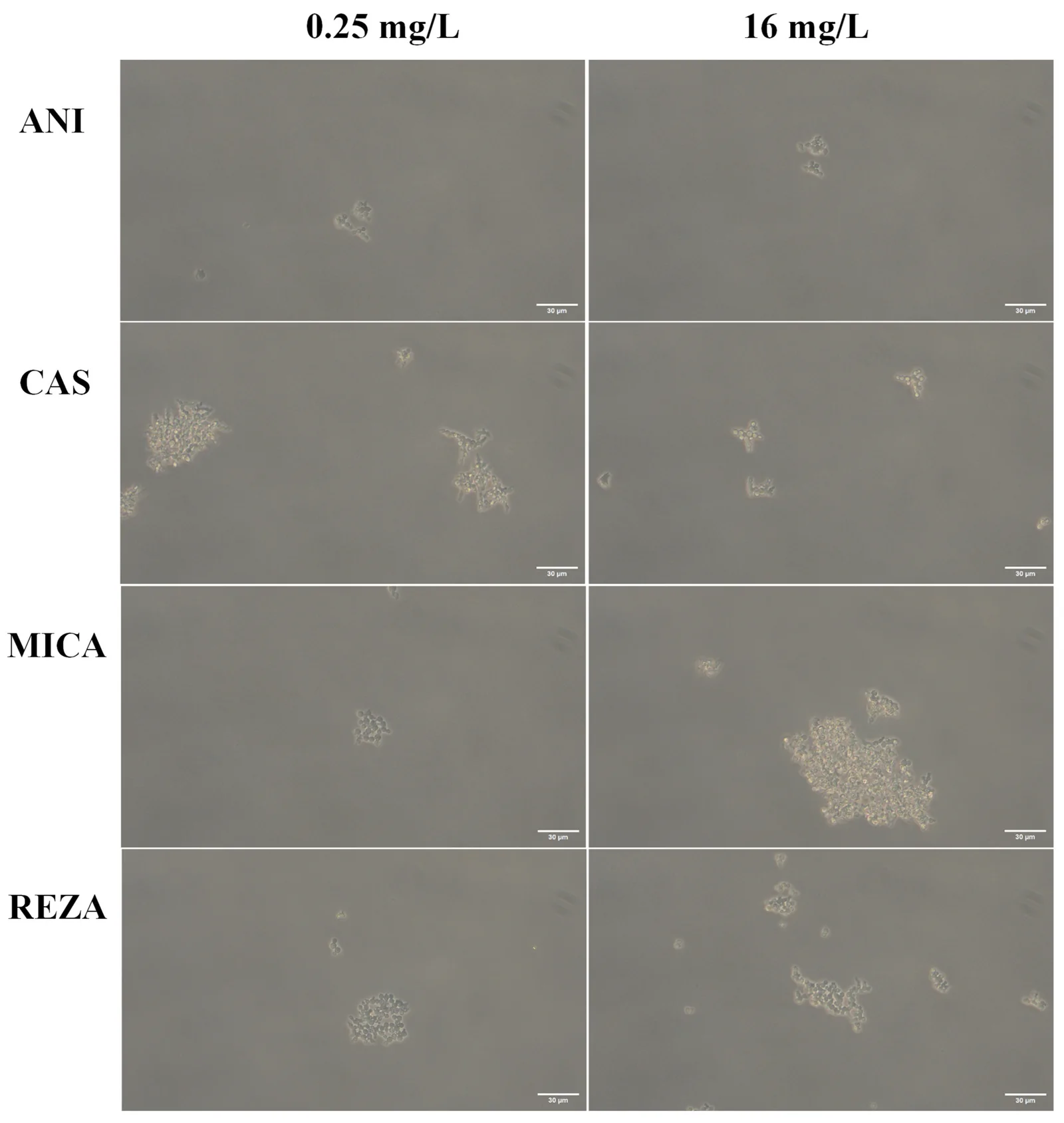
Phase-contrast microscopy images of *C. auris* IFRC4050 cells treated with 0.25 (**left**) and 16 mg/L (**right**) anidulafungin (ANI), caspofungin (CAS), micafungin (MICA) and rezafungin (REZA) in RPMI-1640 at 37 °C. Micrograph was taken after 24 h. Echinocandin-treated fungal cells never produced pseudohyphae. In the case of ANI, small aggregates (~20 µm in diameter) are visible at both concentrations. The largest aggregate (~120 µm diameter) was noticed with MICA at 16 mg/L. Bar: 30 µm. Magnification, ×400.

Pseudohyphae production was detected in all five isolates at both temperatures (Figure 1, Supplemental File S1 and Table 2), but hyphae were never observed. Single elongated cells and pairs were frequently noticed. In the case of isolate MRL40 cca. 66% of fungal cells grew as pseudohyphae at 30 and 37 °C. Pseudohyphae production was the lowest numerically in isolates TMML616 (35.8%) and TMML617 (37.8%) at 30 °C, and statistically non-significant increases were observed with both isolates at 37 °C. Contrastingly, the IFRC4050 strain produced significantly higher numbers of pseudohyphae at 30 °C than at 37 °C (*p* = 0.019). Yeast aggregates were rarely observed. In contrast, echinocandin-treated yeasts at 37 °C showed small rounded (~20 µm) and large (120× ~80 µm) aggregates at both (0.25 and 16 mg/L) echinocandin concentrations (Figure 2). The smallest aggregates (~10 cells) were observed in anidulafungin-treated cells at both concentrations, while more than 100-cell aggregates were seen with micafungin at 16 mg/L (Figure 2). Pseudohyphae were never observed in echinocandin-treated isolates.

### 3.3. Time–Kill Studies

**Figure 3:**
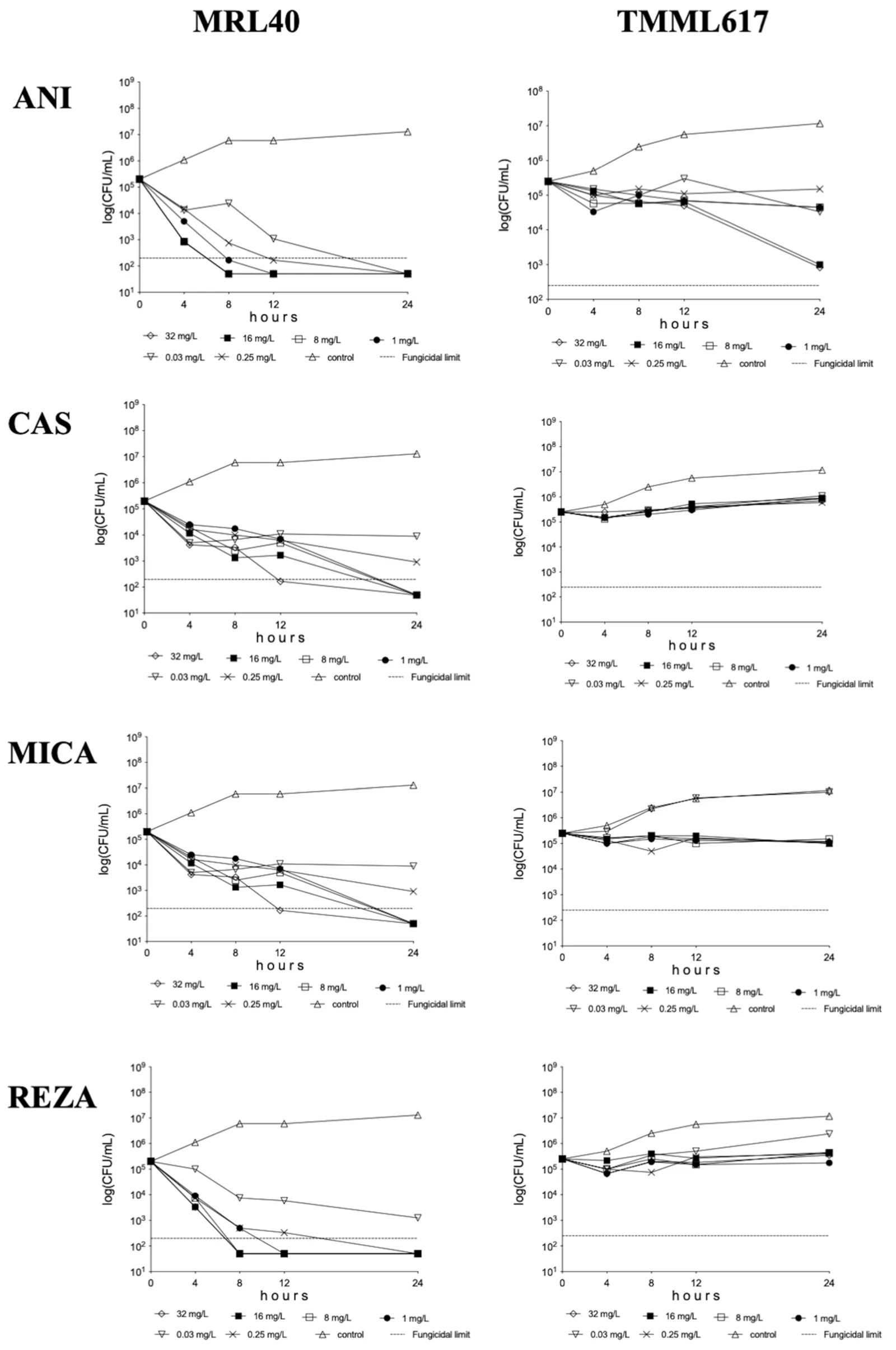
Time–kill plots of anidulafungin (ANI), caspofungin (CAS), micafungin (MICA) and rezafungin (REZA) in RPMI-1640 against isolates MRL40 (**left**) and TMML617 (**right**). Dotted lines indicate the fungicidal limits (≥3 log CFU/mL changes in viable cell count compared to the starting inoculum).

**Figure 4:**
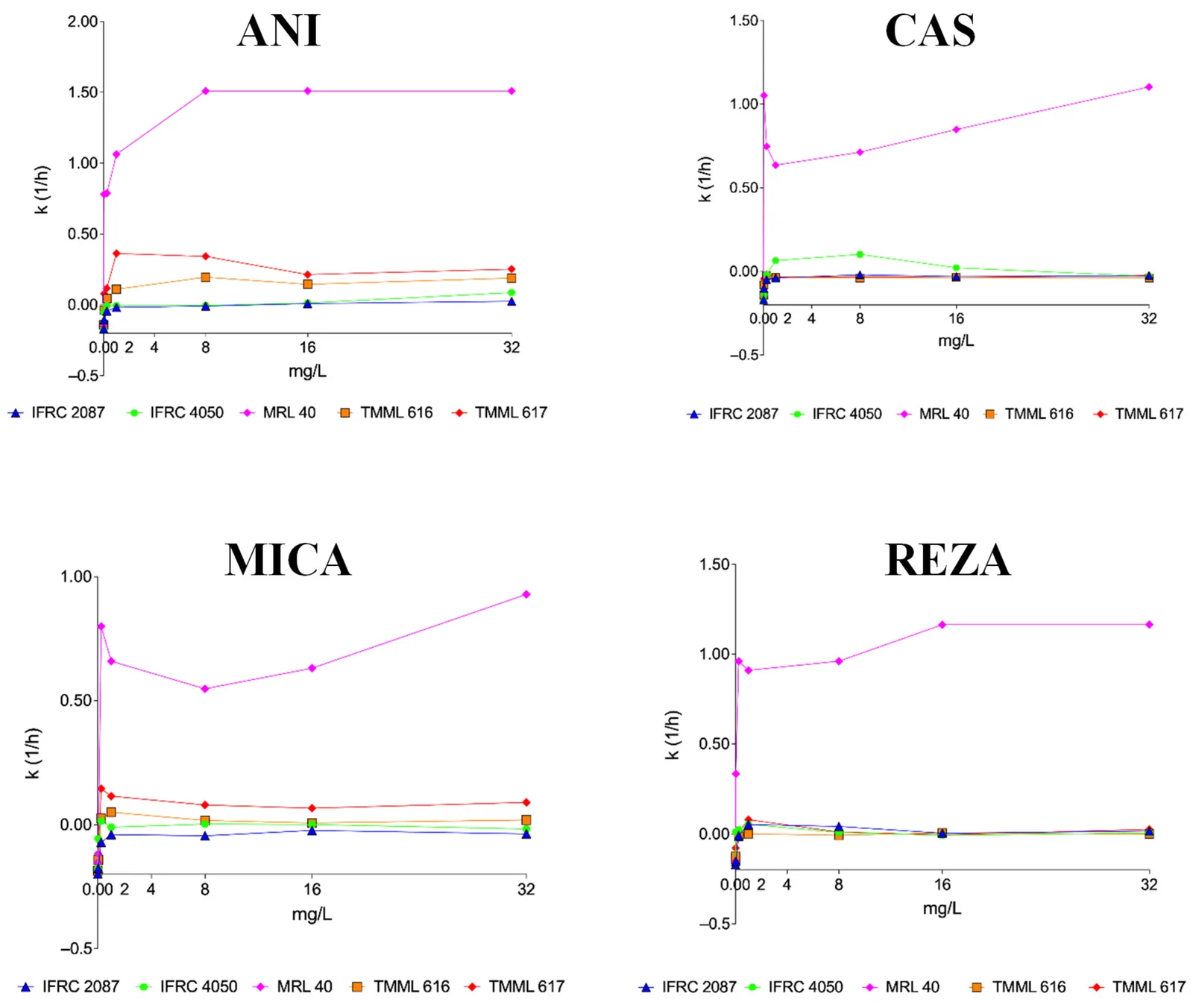
Mean killing rate values of anidulafungin (ANI), caspofungin (CAS), micafungin (MICA) and rezafungin (REZA) in RPMI-1640 against isolates IFRC2087, IFRC4050, MRL40, TMML616 and TMML617. Echinocandin killing rate values (*k* = 1/h) were determined at 0.03, 0.25, 1, 8, 16 and 32 mg/L. Positive and negative *k* values indicate the decrease and increase, respectively, in viable cell numbers.

The four echinocandins showed concentration-dependent killing activities against isolate MRL40 (Figure 3). T99.9 value ranges against isolate MRL40 for anidulafungin at 0.03–32 mg/L, caspofungin at 1–32 mg/L, micafungin at 0.25–32 mg/L and rezafungin at 0.25–32 mg/L were 1.99–3.84 h, 2.72–4.72 h, 3.23–5.48 h and 2.58–3.30 h, respectively. The four echinocandins used against isolates IFRC2087 and IFRC4050 produced <0.5 log CFU decreases, with prominent regrowth and almost always negative *k* values (Figure 4). Against the remaining two isolates (TMML616 and TMML617), anidulafungin and micafungin at ≥0.25 mg/L generated positive *k* values at all tested concentrations (Figure 4); the highest CFU decreases (2.40–2.48 log CFU) were observed at 16–32 mg/L with anidulafungin for isolate TMML617 (Figure 3). For isolates TMML616 and TMML617, caspofungin and rezafungin produced only transient CFU decreases after 4–8 h, followed by regrowth after 24 h (Figure 3); killing rate values were rarely positive (Figure 4). Anidulafungin at 1 and 8 mg/L showed greater (*k* values were 0.36 and 0.34 1/h, respectively) mean killing activities compared to 16 or 32 mg/L (*k* values were 0.21 and 0.25 1/h, respectively) against isolate TMML617 (mini-paradoxical growth) (Figure 4). A similar phenomenon was observed with micafungin against the same isolate (*k* values were 0.15 1/h at 0.25 mg/L, and 0.06 and 0.09 1/h at 16 and 32 mg/L, respectively) [19].

### 3.4. Lethality Experiments

**Figure 5:**
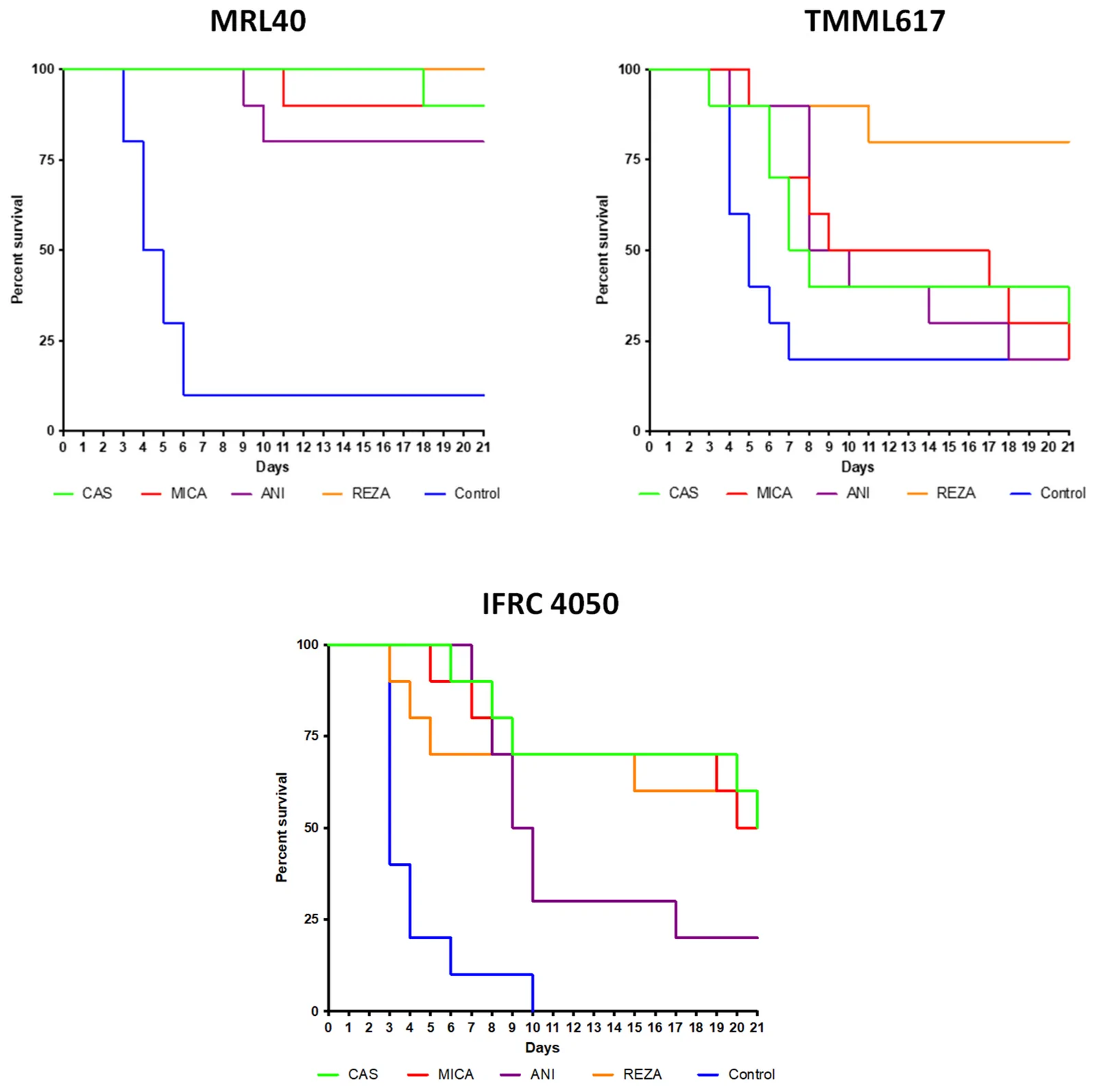
Survival of rezafungin (REZA), anidulafungin (ANI), caspofungin (CAS) and micafungin (MICA)-treated and control neutropenic BALB/c mice infected with the isolates MRL40, TML617 and IFRC4050. The infectious dose was 1 × 10^7^ CFU/mouse. A 20 mg/kg dose of rezafungin was administered on days 1, 3, and 6. Additionally, beginning 24 h post-infection, the mice received 3 mg/kg of caspofungin (Cancidas^®^), 5 mg/kg of micafungin (Mycamine^®^), and 5 mg/kg of anidulafungin (Eraxis^®^) once a day for 6 days. After 21 days, survival rates were compared using the Kaplan–Meier log rank test.

All echinocandin regimens improved the survival in mice infected with the MRL40 strain (*p*-values for anidulafungin, caspofungin, micafungin and rezafungin were 0.0002, 0.0001, 0.0001 and 0.0001, respectively; Figure 5). In contrast, in mice infected with isolate TMML617, only rezafungin significantly improved survival (*p*-values for anidulafungin, caspofungin, micafungin and rezafungin were 0.1606, 0.2121, 0.2286 and 0.0049, respectively; Figure 5). Two micafungin-treated mice infected with isolate TMML617 showed ataxia 2–3 days before their death (died on days 17 and 21). For isolate IFRC4050, all four echinocandins increased survival compared to the untreated control mice (*p*-values for anidulafungin, caspofungin, micafungin and rezafungin were 0.0006, 0.0001, <0.0001 and 0.0006, respectively; Figure 5) and ataxia was not observed.

### 3.5. Fungal Tissue Burden Experiments

**Figure 6:**
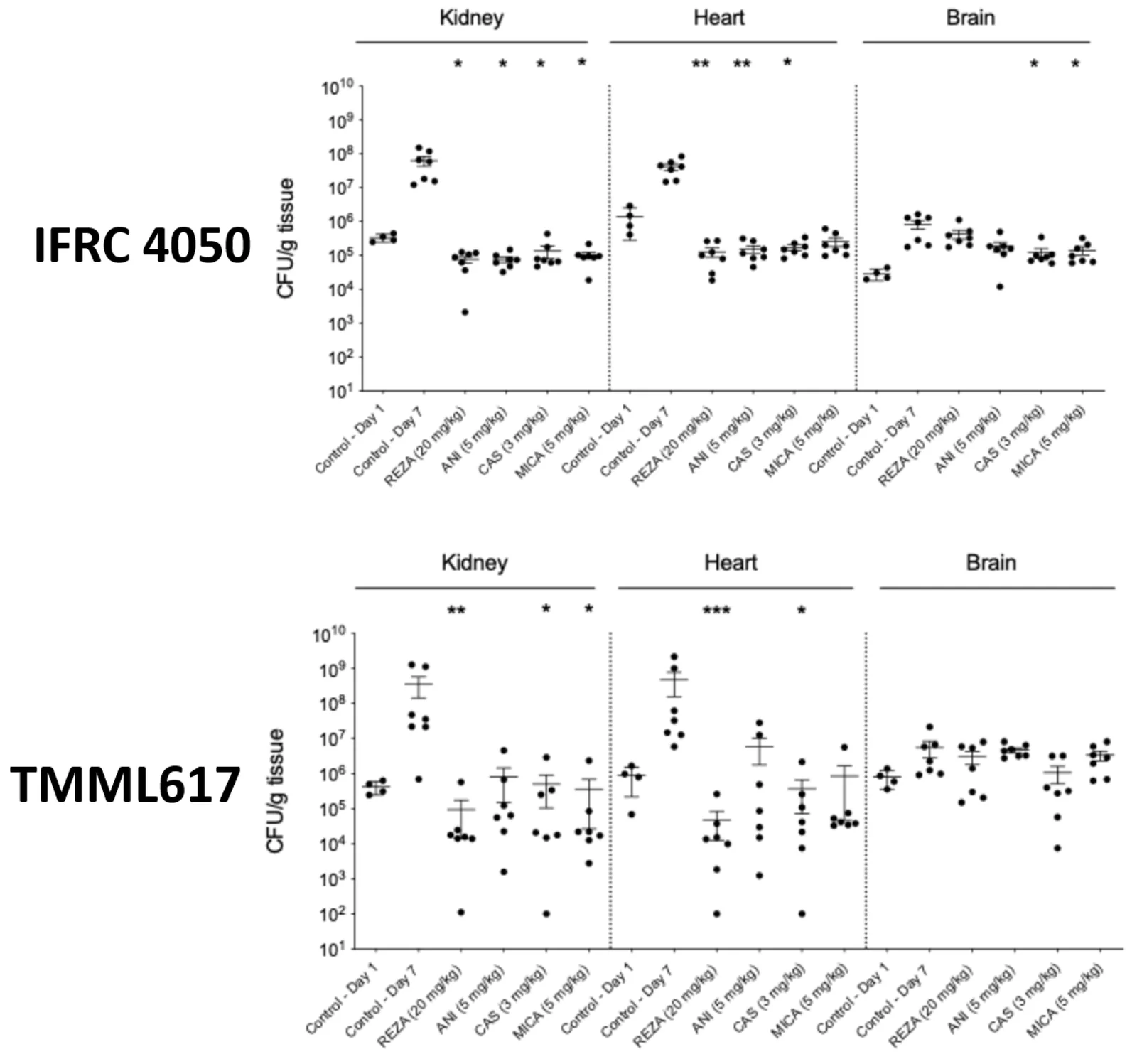

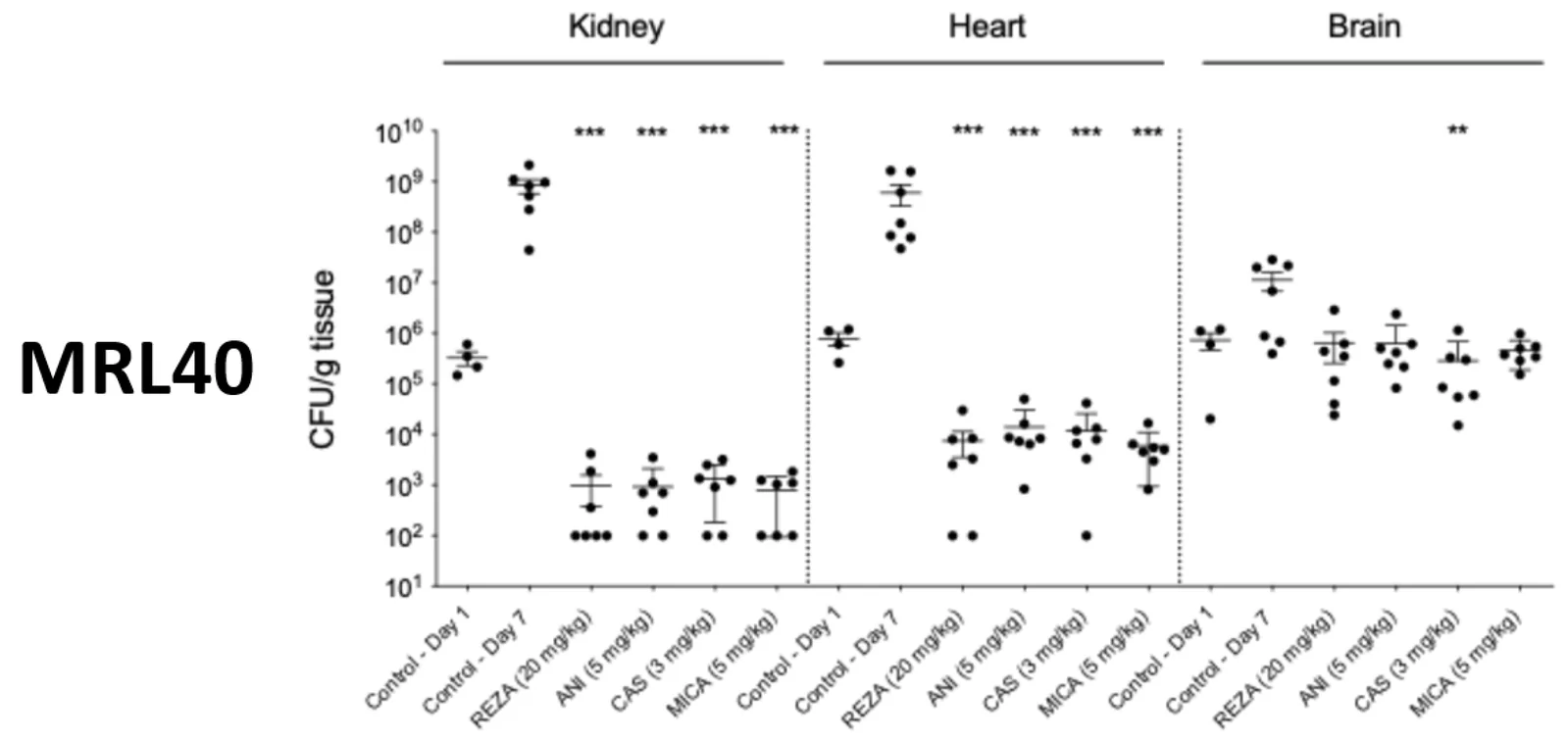
Kidney, heart and brain fungal burdens of rezafungin (REZA), anidulafungin (ANI), caspofungin (CAS) and micafungin (MICA)-treated and control neutropenic BALB/c mice infected with the isolates MRL40, TMML617 and IFRC4050. The infectious dose was 1 × 10^7^ CFU/mouse. A 20 mg/kg dose of rezafungin was administered on days 1, 3, and 6. Additionally, beginning 24 h post-infection, the mice received 3 mg/kg of caspofungin (Cancidas^®^), 5 mg/kg of micafungin (Mycamine^®^), and 5 mg/kg of anidulafungin (Eraxis^®^) once a day for 6 days. The kidney, the heart, and the brain burdens were determined on day 7. The bars represent the medians. Asterisks indicate level of significance (* *p* < 0.05, ** *p* < 0.01, *** *p* < 0.0001).

The four echinocandins produced more than 5 and 4 log mean CFU/gram decreases in the kidneys (*p* < 0.001 for all echinocandins) and hearts (*p* < 0.001 for all echinocandins), respectively, in mice infected with isolate MRL40 compared to control mice on day 7 (Figure 6). Moreover, in the kidneys and hearts, the CFU decreased at least 2 and 1 log CFU/gram, respectively, compared with controls on day 1 with all four echinocandins. Only caspofungin showed statistically significant CFU decreases in the brains (*p* < 0.05). However, in echinocandin-treated mice, the mean fungal burden in brain tissue was always higher than 10^5^ CFU/gram (Figure 6).

In mice infected with isolate TMML617, rezafungin (*p* < 0.01), caspofungin (*p* < 0.05) and micafungin (*p* < 0.05) generated >3 log, >2 log and >2 log CFU/gram decreases, respectively, in the kidneys on day 7. In the hearts, rezafungin (*p* < 0.001) and caspofungin (*p* < 0.05) treatment showed more than 4 and 3 log mean CFU/gram decreases, respectively (Figure 6). However, only rezafungin treatment decreased the tissue burden in the heart and kidneys (at least with 1 log CFU/gram) compared to controls at day 1. None of the echinocandins induced CFU decreases in the brain (*p* > 0.05 for all echinocandins); the mean fungal burden in the brain tissue was higher than 10^6^ CFU/gram (Figure 6).

In mice infected with isolate IFRC 4050, the four echinocandins produced <3 log CFU mean fungal kidney (*p* < 0.05 for all echinocandins) and heart (*p* < 0.01 for rezafungin and anidulafungin, and *p* < 0.05 for caspofungin) burden decreases on day 7 (Figure 6). Caspofungin and micafungin treatments generated statistically significant CFU decreases in the brains (<1 log). However, on day 7 the fungal tissue brain burden in echinocandin-treated mice was higher (~1 log CFU/gram) compared with controls on day 1 (Figure 6).

### 3.6. Histopathology

**Figure 7:**
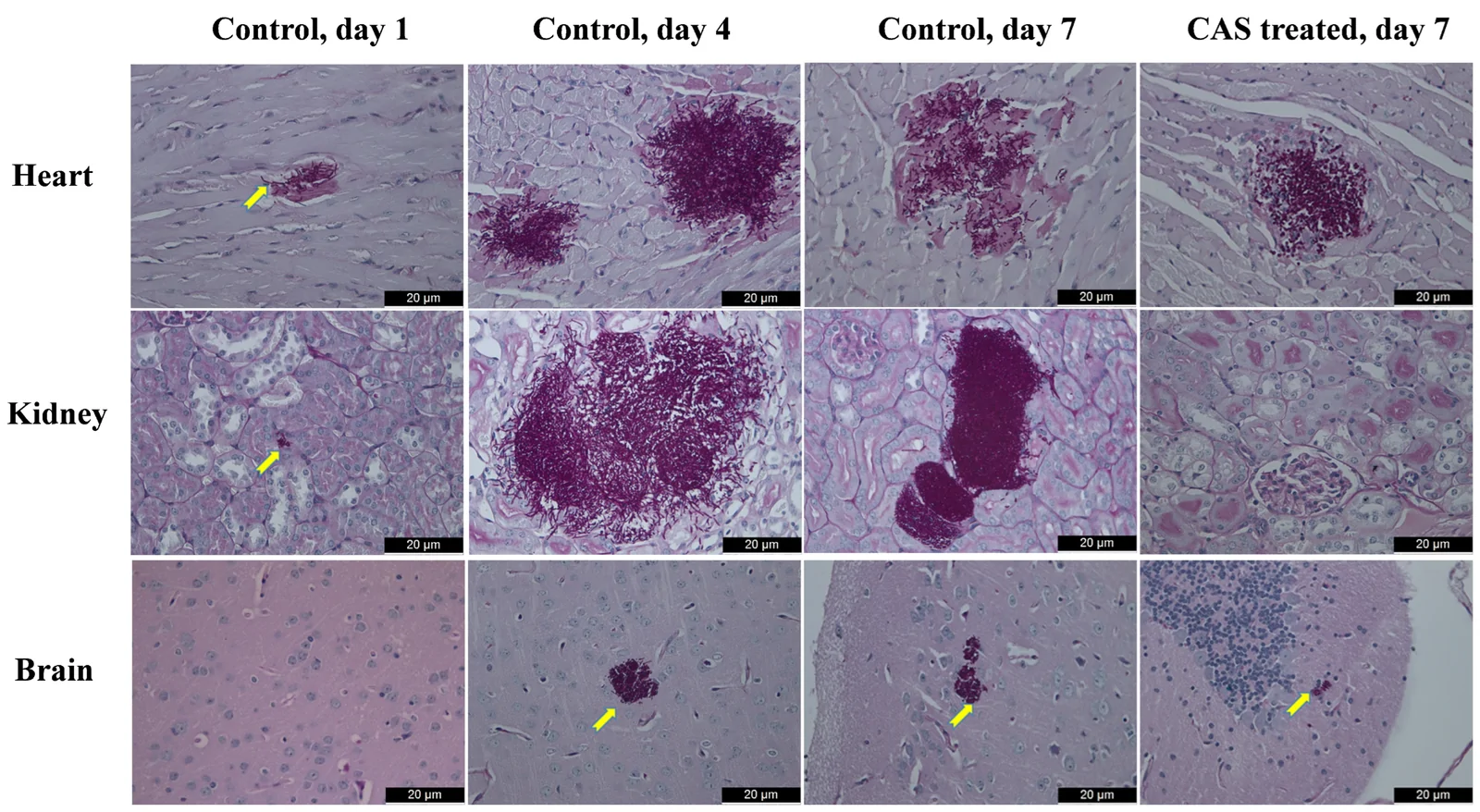
Histopathological findings of the heart, kidney, and cerebrum with periodic acid–Schiff staining in control (days 1, 4 and 7) and caspofungin (CAS)-treated (3 mg/kg once daily for 6 days) mice infected with isolate MRL40. Fungal lesions are indicated by yellow arrows. In control mice, pseudohyphae were visible in the heart and kidneys starting from day 1, but were not detected in the brain. Magnification, ×100.

**Figure 8:**
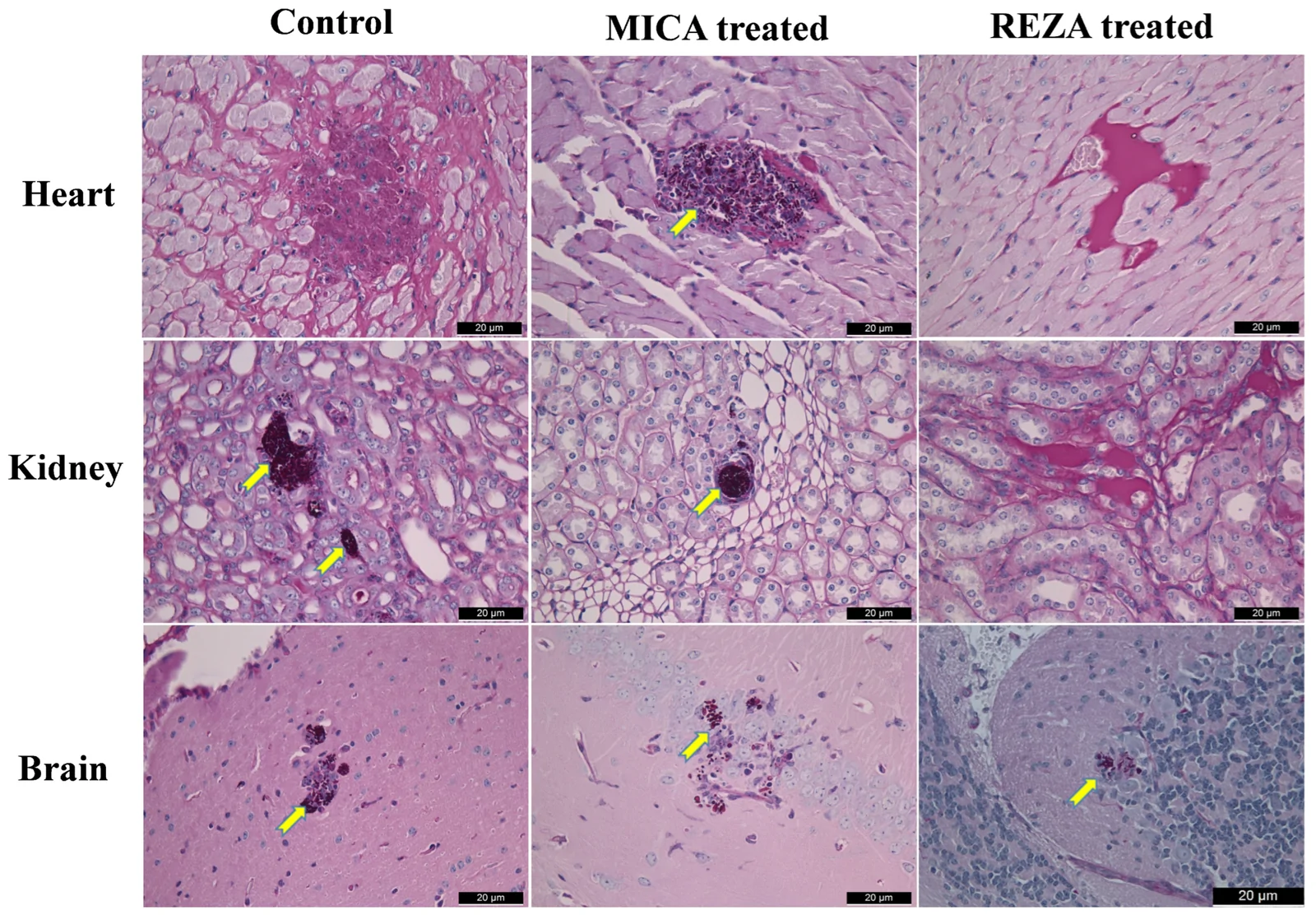
Histopathological examination of the hearts, kidneys and brains using periodic acid–Schiff staining in surviving neutropenic mice infected with isolate MRL40 on day 21. Fungal lesions are indicated by yellow arrows. In the control mouse, scattered fungal cells were observed in the heart within the coagulative necrotic area, but the kidney and brain showed dense aggregates of blastoconidia. In micafungin (MICA)-treated mice (5 mg/kg once daily for 6 days), blastoconidia were visible as one large dense aggregate in the heart, one small dense aggregate in the kidney, and numerous very small aggregates in the brain. In rezafungin (REZA)-treated (20 mg/kg on days 1, 3, and 6) mice, fungal cells were only observed in the brain. Magnification, ×100.

Histopathology showed small and large aggregates of predominantly pseudohyphae in hearts, kidneys and brains in control mice on day 7. Pseudohyphae were visible in the heart and kidneys as early as day 1, but were only detected in the brain from day 4 (Figure 7). The sizes of the aggregates in the heart, kidney and brain were ~20–50, ~50–60 and ~20 µm in diameter, respectively. Echinocandin treatment, especially rezafungin, showed sporadic or no fungal cells in hearts and kidneys, but blastoconidia were always visible in cerebrum or cerebellum. Pseudohyphae were not found in echinocandin-treated mice (Figure 7). The largest aggregate (~40 µm in diameter) was found in the heart in the caspofungin-treated mouse (Figure 7). The heart of the surviving control mouse dissected at the end of the lethality experiment (day 21) showed coagulative necrosis with scattered fungal cells, but large and small aggregates of rounded cells (blastoconidia) were detected in the kidney and brain, respectively. In the echinocandin-treated groups only in the micafungin-treated mouse, large and small aggregates of blastoconidia were observed in the heart and kidney, respectively. However, in the brains, aggregates of blastoconidia (~20 µm in diameter) were always visible in echinocandin-treated mice (Figure 8).

## 4. Discussion

MIC values of the four echinocandins against all five Iranian isolates did not exceed the tentative breakpoints proposed by CDC for anidulafungin, caspofungin and micafungin, or the CLSI susceptible breakpoint of rezafungin [3,22]. In time–kill studies, all four echinocandins showed rapid fungicidal activities against isolate MRL40 and a fungistatic effect with frequently negative killing rate values against the remaining four isolates. A mini-paradoxical effect (decreased killing at higher compared to lower drug concentrations) was noticed for anidulafungin and micafungin against isolate TMML617 (Figure 4). All strains produced pseudohyphae at 30 °C and 37 °C in vitro; isolates IFRC2087, IFRC4050 and MRL40 predominantly grew as pseudohyphae (52.7–65.9%) in RPMI-1640 at both temperatures (Table 2). Pseudohyphae were not detected even with 0.25 mg/L of echinocandins at 37 °C. Our in vivo results correlated with the in vitro killing studies only for isolate MRL40. In the lethality experiments, all echinocandin regimens improved the survival of mice infected with isolates MRL40 and IFRC 4050, but only rezafungin proved to be effective against mice infected with isolate TMML617. The four echinocandins in mice infected with isolate MRL40 induced larger mean CFU/gram decreases in the kidneys and hearts compared to mice infected with isolates TMML617 and IFRC 4050. Fungal growth in the brain, regardless of the isolate tested, was poorly inhibited by echinocandins (Figure 6). Histopathology showed large aggregates of pseudohyphae in hearts, kidneys and brains in control mice (Figure 7). Echinocandin treatment showed sporadic yeast cells in hearts and kidneys (blastoconidia), but yeasts were always visible in the cerebrum or cerebellum. Pseudohyphae were not detected in echinocandin-treated mice (Figure 7 and Figure 8).

Data on echinocandin in vitro killing kinetics and in vivo efficacy against the Iranian *C. auris* clade are lacking. However, Barough and co-workers determined the in vivo pathogenicity of a single Iranian isolate (IFRC4050) and amphotericin B efficacy in an immunocompromised mouse model [25]. They found 60% mortality rate at the end of the 21-day experiment, which was lower compared with our current results with the same isolate (100% mortality after 10 days). In their fungal burden experiments, the highest and lowest fungal load were detected in the hearts and kidneys, and in the brains, respectively, in control mice. Their histopathology analyses indicated that the heart and kidney were the most severely affected organs, followed by the spleen, liver, lung, and brain [25]. The lack of studies evaluating echinocandin activity against Iranian clade V *C. auris* precludes comparative discussion of our results.

A notable strength of this study is that the in vitro efficacy of the four approved echinocandins was tested against the recently described Iranian isolates. Pseudohyphae were visualized in all five isolates with phase-contrast microscopy in RPMI-1640 at 30 and 37 °C. A limitation of this work is that in vivo experiments were not performed with all five isolates, although all four approved echinocandins were tested against one of the two Iranian isolates derived from different cities (Babol and Shiraz) in vivo. Histopathology also revealed pseudohyphae in the kidneys, heart and brain in mice infected with the three isolates from the three different Iranian cities. The fungal burden was determined only in the kidneys, heart and brain, which may be regarded as another limitation. However, other authors, as well as our previous studies, indicated that the heart and kidneys are the most important target organs in cases of *C. auris* candidemia [23,24,25].

*C. auris* was considered a species that produces cellular aggregates in vitro but does not produce pseudohyphae either in vitro nor in vivo [26,27]. Other authors found that changes in the environmental conditions and genotoxins (hydroxyurea, methyl methane sulfonate and 5-fluorocytosine) could provoke pseudohyphae formations [28]. Yue et al. observed filament formation after passage in the mouse model with the four main clades [29]. In a recent study, Gifford et al. developed a fish embryo yolk-sac microinjection model using *Aphanius dispar* (Arabian killifish) at human body temperature to study the virulence of five *C. auris* clades [30]. In their experimental conditions, clades I-IV showed budding yeast morphologies both in vitro and in vivo, but filamentation was observed with the Iranian isolate in vivo. They found up-regulation of genes responsible for filamentation and biofilm formation, including UME6 (Upstream Multi-Environment 6), HGC1 (hypha-specific G1 cyclin-dependent protein serine/threonine kinase) and *C. auris*-specific SCF1 (Surface Colonization Factor 1). These gene expression increases aid in developing biofilm on abiotic (i.e., medical device) surfaces and are essential for colonizing the skin. Moreover, interaction of Scf1 protein with other adhesins (i.e., ALS4112) promotes fungal cell aggregation. It is noteworthy that all the publicly available and published Iranian isolates in our study produced pseudohyphae in vitro in RPMI-1640 medium. As RPMI-1640 medium is not considered a stressor to *C. auris*, pseudohyphae production seems to be an innate ability of Iranian clinical isolates. Moreover, isolate IFRC2087 in an Arabian killifish model [30], and isolates IFRC4050, MRL40 and TMML617 in our neutropenic mouse model, showed pseudohyphae formation in vivo.

Cosio et al. demonstrated pseudohyphal growth in the clade IV strain CDC B11903 in RPMI 1640 supplemented with 10% fetal bovine serum at 25 °C, 37 °C and 42 °C for 24 h in light microscopy [31]. Pseudohyphal growth was observed only in single *C. auris* cells at each temperature, suggesting that pseudohyphae production is a rare event, and temperature did not substantially affect the capacity of this strain to undergo pseudohyphal growth [31]. In our study, the pseudohyphae production was universal for the tested isolates and temperature hardly had an influence (Figure 1). However, for isolate IFRC2087, a significantly higher number of pseudohyphae were observed at 30 °C than at 37 °C. Because *C. auris* cells were cultured without agitation, we cannot rule out that the pseudohyphae formation was originally higher than we measured. Single and double elongated cells were frequently found in the microscopic fields, suggesting release of these cells by the mechanical damage of pseudohyphae when the fungal solution was removed from the tubes with a pipette (Figure 1).

In vivo efficacy against the Iranian isolates was echinocandin- and isolate-dependent. Although all four echinocandins were highly effective against isolate MRL40 both in vitro and in vivo, the in vitro and in vivo correlation was poor for the other two isolates. Despite the weak fungistatic activity of the four echinocandins against isolate IFRC4050, all four antifungals significantly increased survival and decreased fungal tissue burdens. In contrast, anidulafungin produced the largest CFU reduction in the killing study for isolate TMML617, but showed very poor activity in the lethality and fungal tissue burden experiments. Interestingly, histopathology showed no fungal cells in these echinocandin-treated mice. It is notable that against isolates TMML617 both anidulafungin and micafungin produced better killing activities at lower compared to higher concentrations (mini-paradoxical growth). Echinocandin exposure induces a variety of stress adaptation pathways with an increased cell wall chitin amount coupled with aggregate formation in the internal organs, which may explain the weak in vivo efficacy of anidulafungin, caspofungin and micafungin against some Iranian isolates [32].

With the exception of isolate MRL40, rezafungin showed weak in vitro fungistatic activity against the Iranian isolates belonging to *C. auris* clade V. In contrast, rezafungin had similar or better in vivo activity than anidulafungin, caspofungin, and micafungin against the three isolates from different geographical areas in Iran. It is notable that only rezafungin was efficacious in the lethality experiment in mice infected with isolate TMML617. Our current results align with previous observations that correlations do not always exist between in vitro activity and in vivo efficacy [19,24]. A possible explanation for the increased in vivo efficacy of rezafungin against the *C. auris* isolates is its differentiated pharmacokinetic profile (i.e., front-loaded dosing rapidly achieves high concentrations in plasma and tissues) [15,16].

It is well known that the South Asian, South African and South American clades are frequently associated with invasive, life-threatening infections and large outbreaks, while the East Asian clade is mainly responsible for ear infections. The fact that three isolates from Korea related to bloodstream infections proved to be genetically similar to isolates producing ear infections suggests that, in special clinical situations, the East Asian clade could become invasive [33]. The same outcome may be true for the fifth *C. auris* clade, as its virulence in our study was comparable with that of the previously described four main clades [23,24]. The special microenvironment of the intact skin or external ear allows pseudohyphae to be produced in Iranian isolates at 30 °C. In colonized patients, disruption of the integrity of the skin or mucous membrane may lead to invasion of the deeper tissues and blood due to pseudohyphae production at 37 °C, as described in the present study. Alarmingly, a report of the sixth isolation of *C. auris* clade V in Iran, from a patient with acute myeloid leukemia, was just published [34]. The strain was found to be closely related to IFRC4050, with a difference of 11 SNPs, and the isolate showed high MICs to anidulafungin and caspofungin (≥16 mg/L for both echinocandins) without any mutations in *FKS1*, raising the possibility of emergence of echinocandin resistance in this clade. The authors underline the importance of genomic surveillance of *C. auris* isolates in Iran to track the emergence and spread of new resistance mechanisms and to understand the population dynamics. Importantly, the therapeutic choices against this isolate were significantly narrowed, leading to a further public health threat [34].

In this study, isolates of the fifth clade without echinocandin exposure universally showed strong pseudohypha production both in vitro and in vivo. However, in the presence of echinocandins, isolates belonging to the fifth clade behaved similarly to clades I-IV, producing large and small aggregates of blastoconidia in vitro and in vivo [23,24]. Our current and previous in vivo results suggest that rezafungin, and to a lesser extent caspofungin, regardless of the *C. auris* clade, are highly effective in the treatment of invasive fungal infections, including myocarditis and pyelonephritis due to *C. auris* [24] when echinocandin MIC values are low.

## Figures and Tables

**Table 1:** MIC (mg/L) values of anidulafungin (ANI), caspofungin (CAS), micafungin (MICA) and rezafungin (REZA) in RPMI-1640 against *C. auris* Iranian isolates, and *C. auris*-type strain (CBS 10913) and *C. parapsilosis* ATCC 22019 strain. MICs were determined three times using the CLSI broth microdilution method.

Strain	Place	Body Site	ANI	CAS	MICA	REZA
IFRC2087 (CBS 18598)	Babol	ear	≤0.03	0.5	≤0.03	0.12–0.25
IFRC4050 (CBS 18600)	Babol	ear	0.12	0.12	≤0.03	0.12
MRL40 (CBS18599)	Isfahan	ear	≤0.03	0.06	≤0.03	0.06
TMML616 (CBS18601)	Shiraz	skin	≤0.03	0.25	0.12	0.12
TMML617 (CBS18602)	Shiraz	ear	≤0.03	0.25	0.12	0.12
*C. auris*-type strain	Japan	ear	≤0.03	0.5	0.06	0.12
ATCC 22019	-	-	0.5	0.5	0.5	0.5

**Table 2:** The relative frequency of pseudohyphae production among isolates belonging to the Iranian clade at 30 °C and 37 °C in RPMI-1640 medium. Pseudohyphae formation was defined as at least three (≥3) elongated fungal cells in chain. The number of cells in pseudohyphae was divided by the total number of cells counted in the case of each isolate. Pseudohyphae production at 30 °C and 37 °C was compared using the Mann–Whitney U-test in each isolate.

Isolates	Percentages of Cells in Pseudohyphae Compared to Total Cell Number (Number of Cells in Pseudohyphae/Total Cell Number)		Significance
	30 °C	37 °C	
IFRC2087	60.2% (1744/2899)	52.7 (880/1670)	*p* = 0.019
IFRC4050	55.5 (1089/1964)	62.3 (999/1603)	*p* = 0.283
MRL40	65.9 (1141/1729)	65.9 (987/1496)	*p* = 0.696
TMML616	35.8 (704/1965)	39.7 (565/1422)	*p* = 0.682
TMML617	37.8 (902/2384)	43.5 (839/1928)	*p* = 0.39

## Data Availability

The original contributions presented in this study are included in the article. Further inquiries can be directed to the corresponding author.

## Supplementary Materials

The following supporting information can be downloaded at: https://www.mdpi.com/article/10.3390/jof12080552/s1, Figure S1: Phase-contrast microscopy images of *C. auris* cells in RPMI-1640 with isolate IFRC2087 at 30 °C after 24 h incubation. Magnification, ×200; Figure S2: Phase-contrast microscopy images of *C. auris* cells in RPMI-1640 with isolate IFRC2087 at 37 °C after 24 h incubation. Magnification, ×200; Figure S3: Phase-contrast microscopy images of *C. auris* cells in RPMI-1640 with isolate IFRC4050 at 30 °C after 24 h incubation. Magnification, ×200; Figure S4: Phase-contrast microscopy images of *C. auris* cells in RPMI-1640 with isolate IFRC4050 at 37 °C after 24 h incubation. Magnification, ×200; Figure S5: Phase-contrast microscopy images of *C. auris* cells in RPMI-1640 with isolate MRL40 at 30 °C after 24 h incubation. Magnification, ×200; Figure S6: Phase-contrast microscopy images of *C. auris* cells in RPMI-1640 with isolate MRL40 at 37 °C after 24 h incubation. Magnification, ×200; Figure S7: Phase-contrast microscopy images of *C. auris* cells in RPMI-1640 with isolate TMML616 at 30 °C after 24 h incubation. Magnification, ×200; Figure S8: Phase-contrast microscopy images of *C. auris* cells in RPMI-1640 with isolate TMML616 at 37 °C after 24 h incubation. Magnification, ×200; Figure S9: Phase-contrast microscopy images of *C. auris* cells in RPMI-1640 with isolate TMML617 at 30 °C after 24 h incubation. Magnification, ×200; Figure S10: Phase-contrast microscopy images of *C. auris* cells in RPMI-1640 with isolate TMML617 at 37 °C after 24 h incubation. Magnification, ×200.
