# Supplementary material for: In Vitro Activity and In Vivo Efficacy in a Murine Model of Rezafungin, Anidulafungin, Caspofungin, and Micafungin Against the Fifth Clade of Candida (Candidozyma) auris
Source: J Fungi (Basel). 2026 Jul 24;12(8):552. doi: 10.3390/jof12080552 (PMC13514925; doi:10.3390/jof12080552)
Supplement: Supplementary file 1 [file jof-12-00552-s001.zip › Figure legends and Images for Supplement 1.pdf]

Supplemental File S1.

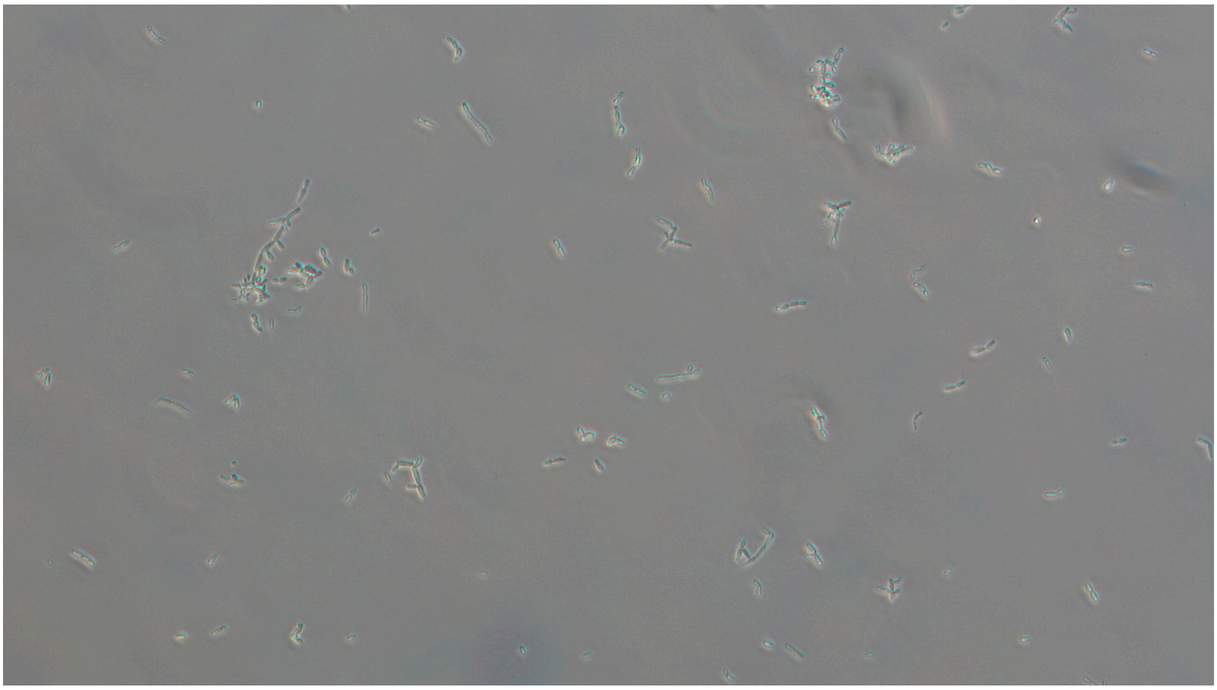

Figure S1. Phase-contrast microscopy images of *C. auris* cells in RPMI-1640 with isolate IFRC2087 at 30 °C after 24 hours incubation. Magnification, ×200.

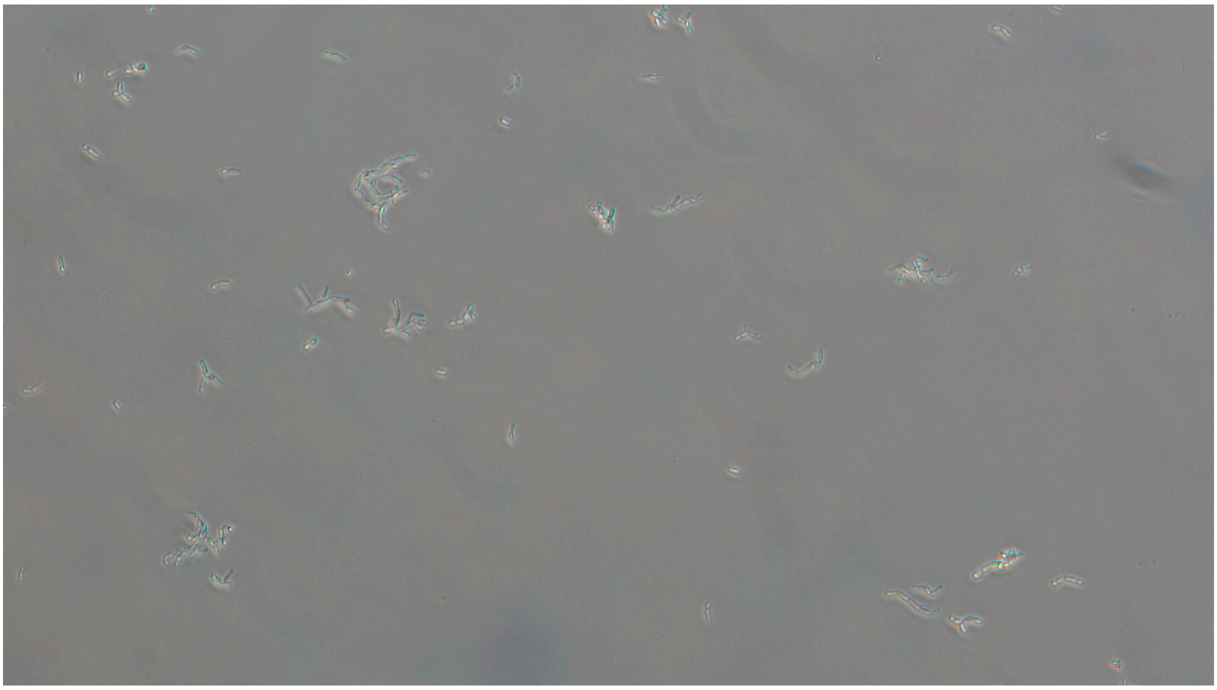

Figure S2. Phase-contrast microscopy images of *C. auris* cells in RPMI-1640 with isolate IFRC2087 at 37 °C after 24 hours incubation. Magnification, ×200.

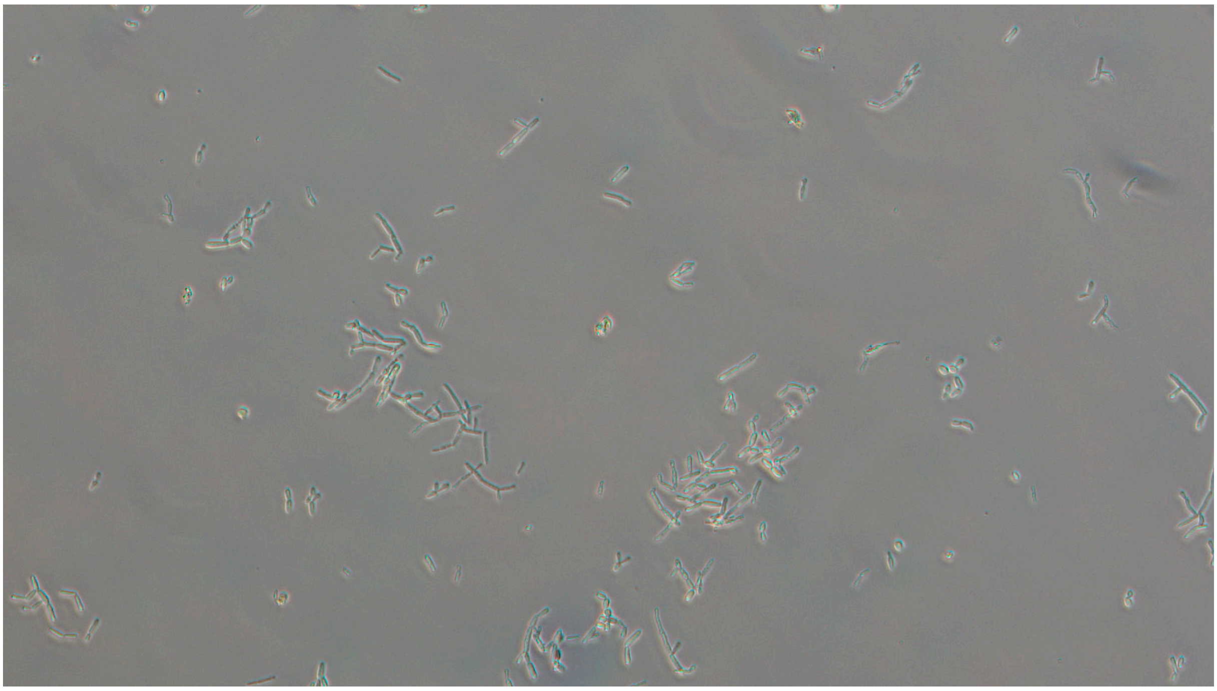

Figure S3. Phase-contrast microscopy images of *C. auris* cells in RPMI-1640 with isolate IFRC4050 at 30 °C after 24 hours incubation. Magnification, ×200.

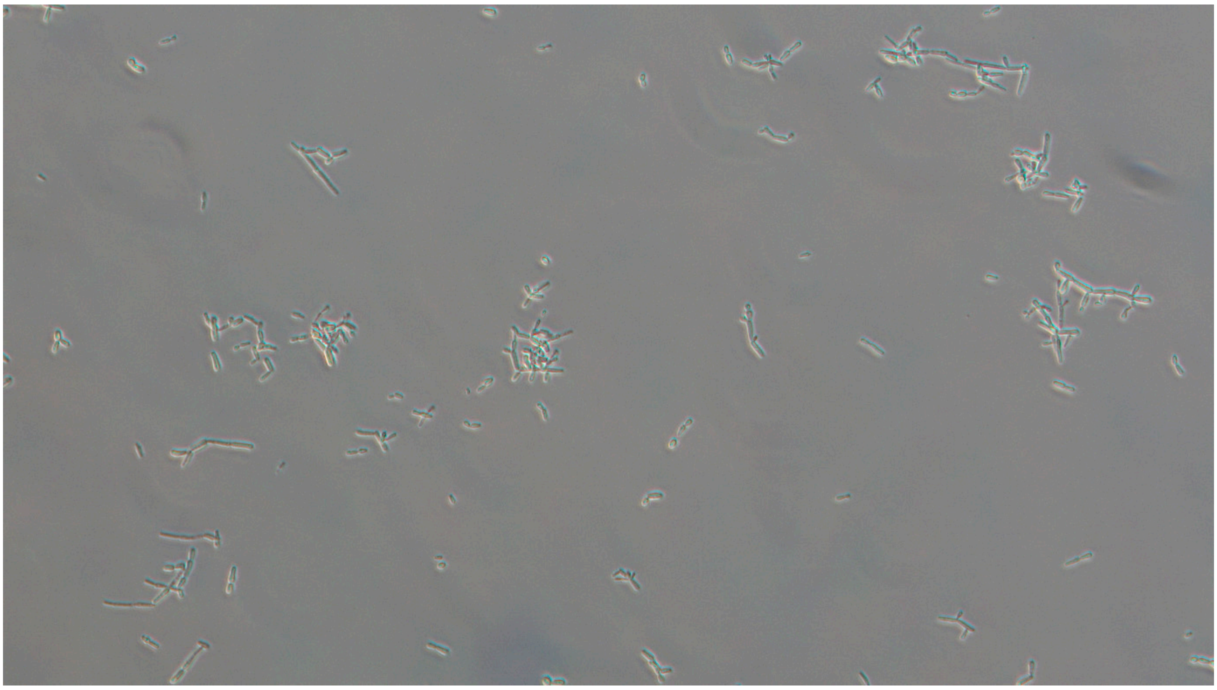

Figure S4. Phase-contrast microscopy images of *C. auris* cells in RPMI-1640 with isolate IFRC4050 at 37 °C after 24 hours incubation. Magnification, ×200.

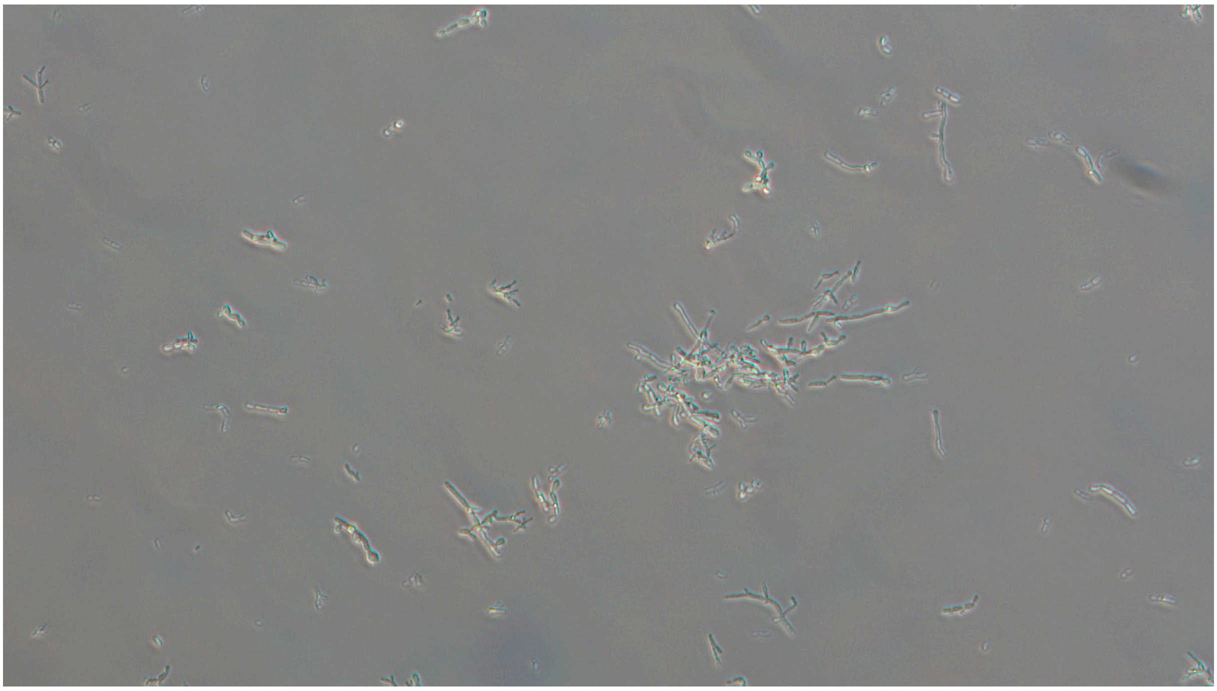

Figure S5. Phase-contrast microscopy images of *C. auris* cells in RPMI-1640 with isolate MRL40 at 30 °C after 24 hours incubation. Magnification, ×200.

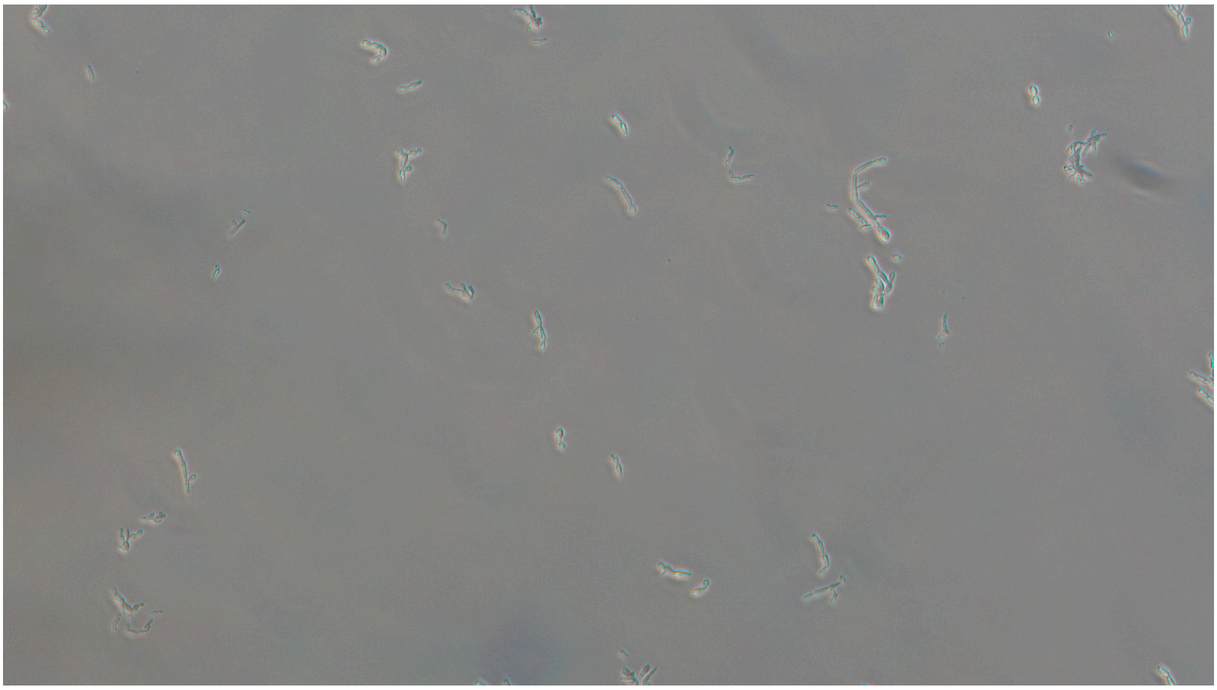

Figure S6. Phase-contrast microscopy images of *C. auris* cells in RPMI-1640 with isolate MRL40 at 37 °C after 24 hours incubation. Magnification, ×200.

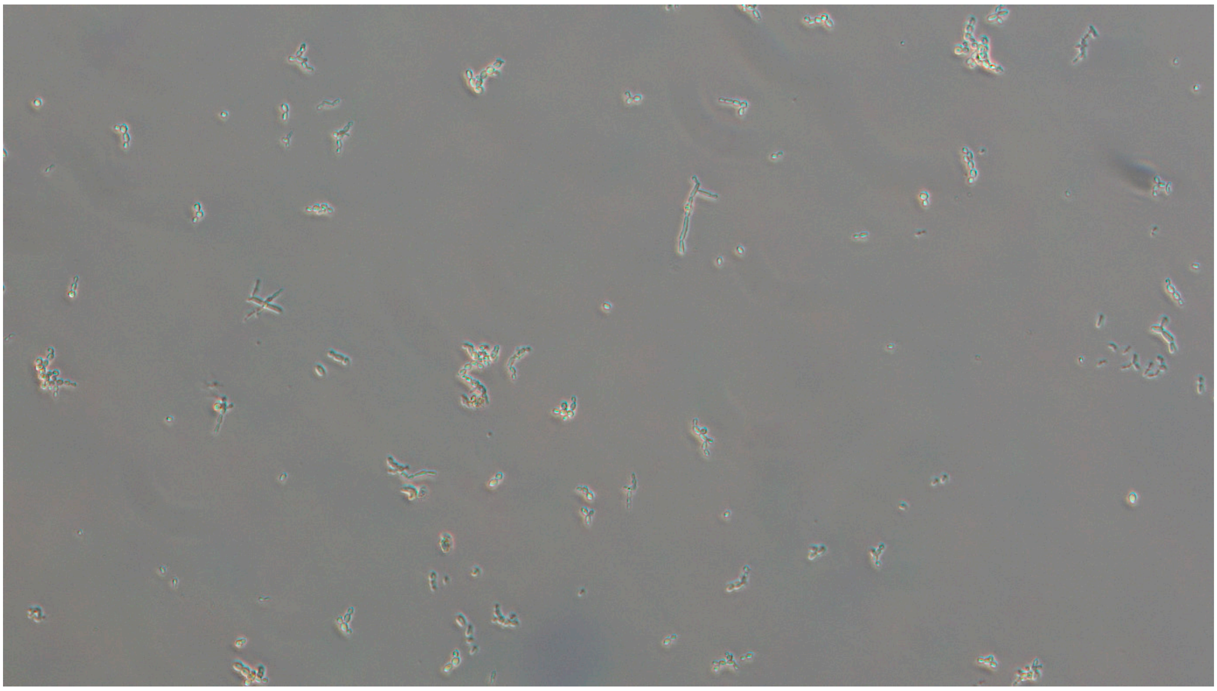

Figure S7. Phase-contrast microscopy images of *C. auris* cells in RPMI-1640 with isolate TMML616 at 30 °C after 24 hours incubation. Magnification, ×200.

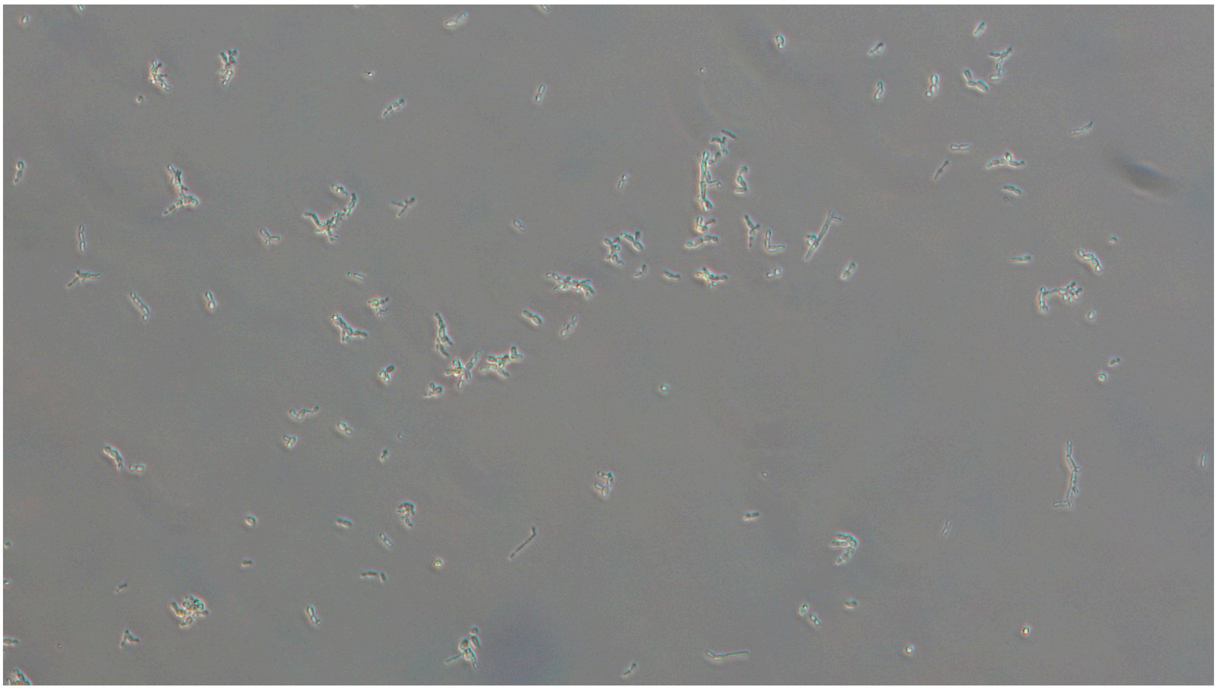

Figure S8. Phase-contrast microscopy images of *C. auris* cells in RPMI-1640 with isolate TMML616 at 37 °C after 24 hours incubation. Magnification, ×200.

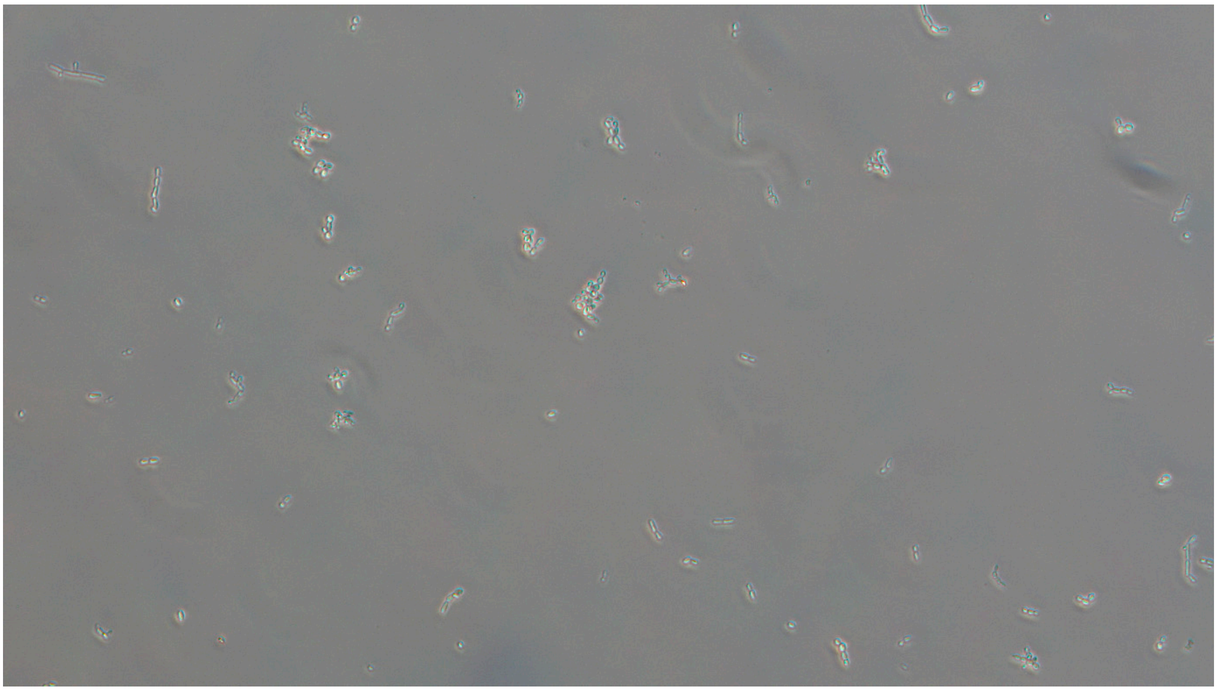

Figure S9. Phase-contrast microscopy images of *C. auris* cells in RPMI-1640 with isolate TMML617 at 30 °C after 24 hours incubation. Magnification, ×200.

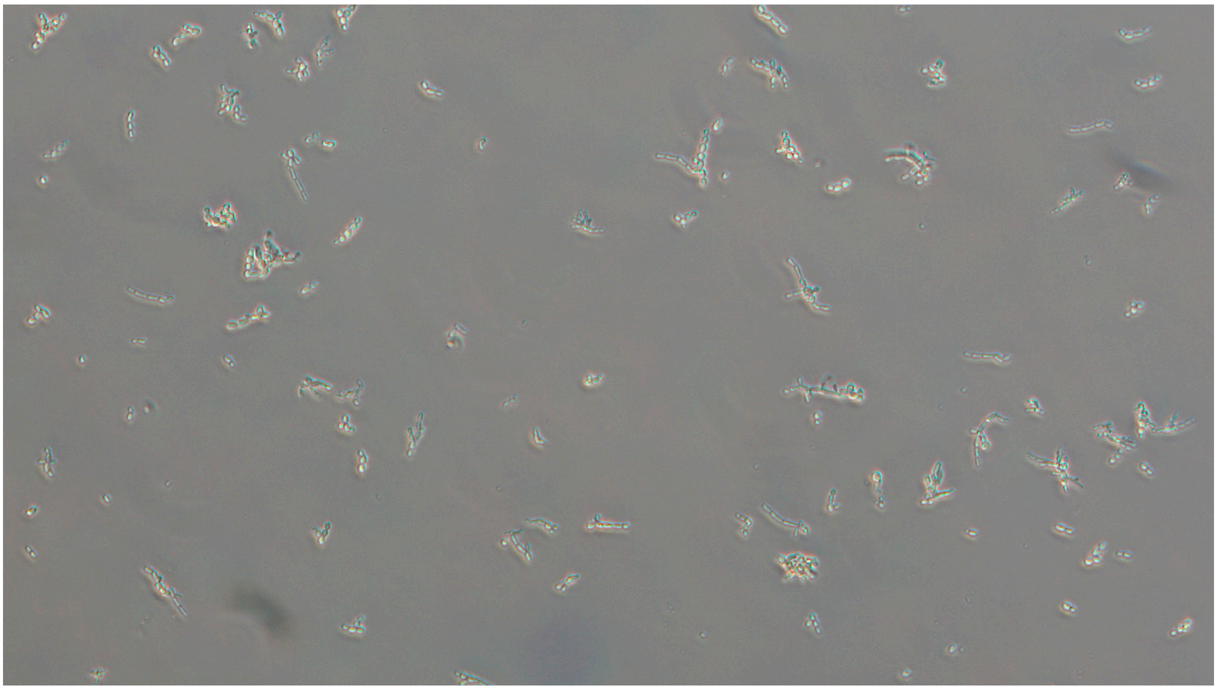

Figure S10. Phase-contrast microscopy images of *C. auris* cells in RPMI-1640 with isolate TMML617 at 37 °C after 24 hours incubation. Magnification, ×200.
